# Heavy metals and metalloids exposure and *in vitro* fertilization: Critical concerns in human reproductive medicine

**DOI:** 10.3389/frph.2022.1037379

**Published:** 2022-11-21

**Authors:** Cecilia Nwadiuto Obasi, Chiara Frazzoli, Orish Ebere Orisakwe

**Affiliations:** ^1^ Department of Experimental Pharmacology & Toxicology, Faculty of Pharmacy, University of Port Harcourt, Port Harcourt, Nigeria; ^2^ Department for Cardiovascular, Dysmetabolic and Aging-Associated Diseases, Istituto Superiore di Sanità, Rome, Italy; ^3^ African Centre of Excellence for Public Health and Toxicological Research (ACE-PUTOR), University of Port Harcourt, Port Harcourt, Nigeria

**Keywords:** heavy metals and metalloids, toxicological concerns, *in vitro* fertilization, human reproductive medicine, pregnancy rate, public health

## Abstract

Exposures to heavy metals and metalloids have been associated with decreased fecundity and fertility in couples conceiving *via* assisted reproduction. Heavy metals and metalloids can alter the homeostasis of critical hormones controlling sexual maturation by binding to critical hormones and receptors. This may disrupt the time course of sexual maturation directly or indirectly affecting reproductive competence in males and females. The present review aims to provide a summarized overview of associations between heavy metal exposure, reproductive concerns, and IVF outcomes. A systematic review was conducted according to the Preferred Reporting Items for Systematic Reviews and Meta-Analyses (PRISMA) in Google Scholar, Scopus, EMBASE and PubMed databases. Initial search produced 1,351 articles from which 30 articles were eligible to be included in the systematic review. From our results, 16 articles reported associations between selected heavy metals and IVF outcomes, while 14 articles summarized the role of heavy metals in reproductive concerns. For the studies on IVF outcomes, different human samples were examined for heavy metals. Heavy metals and metalloids (Pb, Hg, Cd, Cr, Mn, As) correlated negatively with oocyte fertilization/pregnancy rates in hair, follicular fluid, serum, urine and seminal plasma samples, while Cd and Hg in whole blood samples showed no associations. For the studies on reproductive concerns, high levels of heavy metals/metalloids were implicated in the following conditions: infertility (Cd, Pb, Ba, U), spontaneous abortion/miscarriage (Pb, Cd, Sb), congenital heart disease (Al, Mg, Cd), PCOS (As, Cd, Hg, Pb), endometriosis (Pb) and uterine leiomyomata (Hg). Taken together, the results of our study suggest that the impact of heavy metals and metalloids exposure on reproductive health may contribute to the failure rates of *in vitro* fertilization.

## Introduction

Environmental heavy metals and metalloids pollution has been considered as one of the major challenges affecting assisted reproductive medicine in humans. Couples residing in residential areas of low environmental toxicants levels show more positive IVF outcomes (1). Couples residing in industrial areas with high contaminant levels, have less IVF success rates implying that environmental factors may have some adverse effects on IVF outcome (2).

Heavy metals and metalloids are known to possess high atomic numbers and densities of five times above the density of water (3). These are often grouped into essential (manganese, copper, chromium, zinc, selenium, etc) and non-essential metals (lead, cadmium mercury etc). Essential metals and metalloids play significant beneficial role in human health management and other living things at the bio-permissible levels set by standard regulatory bodies (4, 5). These include manganese, copper, chromium, zinc, selenium and others. Some of them are critical components of many metabolic regulating enzymes and signalling pathways. However, when concentration exceeds regulatory limits, biotoxicity ensues impacting on different organs and systems in biological systems (4, 5). The metals and metalloids classified as non-essential heavy metals and metalloids are toxic to biological systems even at trace levels (6). These include lead, arsenic, cadmium, chromium, mercury etc.

The mechanism underlying heavy metal toxicity in humans is mainly due to their interaction with the sulfhydryl (SH) enzyme groups in the non-enzymic antioxidant systems and subsequent inhibition of these enzymes required in energy generation in many metabolic and signal transduction pathways (7). Heavy metals preferentially replace H atom from the sulphydryl groups on the reduced glutathione moieties resulting to the formation of organo–metallic complexes with potentials of deactivating any further biochemical reactions (8).

Human gametes are said to be sensitive and error prone. Exposure to heavy metals and metalloids in the environment can further exacerbate this process directly or indirectly affecting gametogenesis, alter fertilization, depriving the embryo with essential nutrients and lower overall implantation rate in the uterine mucosa (9).

*In vitro* fertilization (IVF) is a type of Assisted Reproductive Technology (ART). It is the most effective and commonly used procedure to address issues of infertility especially in humans (10–12). The process involves multi-step medical procedures in which a matured oocyte (egg) from the ovaries of a female is collected and externally fertilized by sperm cells from a male and then implanted into the uterus of the female after undergoing embryo culture for two-seven days (13). The intention of this procedure is generally to establish pregnancy through an orchestrated sequence of events such as stimulation, egg retrieval, insemination, embryo culture and transfer (11, 12). Since its inception in 1978, IVF has steadily increased and has undergone significant transformation in many ways in aiding women to achieve pregnancy. An estimated 8–10 million children have been born through IVF and other types of ART (14). The success rate of this process has been determined by some factors which include age of the mother, cause of sterility, integrity of the fetus, and lifestyle. Overtime, the technology has expanded to also improve on the success rate of women who consider IVF as an alternative to conceive (12, 15).

Irrespective of the tremendous feat achieved in IVF technology, environmental heavy metals and metalloids pollution continue to pose significant threat on the integrity of the embryo cultured for implantation. Quality of the environment plays a significant role on the success rate of this technology in reproductive medicine. Environmental quality has been implicated in poor ovarian stimulation and response especially in women preparing for IVF (16). Metals and metalloids may restrain morphology of gametes resulting in the formation of poor-quality zygote with diminished viability in cell division (17). Environmental heavy metals and metalloids may cause retardation in cleavage rate of the zygote into a blastocyte with an attendant failure in implantation or recurrent miscarriages. Recurring pregnancy failures following repeated IVFs are often associated with psychological and emotional burden (18). Many reproductive endocrinologists are unaware of the influence of heavy metals and metalloids on the success of IVF treatments that may have surprisingly contributed to prevalence of women who drop out of IVF cited consistently in studies conducted across the globe (10, 19).

Prevalence and persistence nature of heavy metals and metalloids in the environment have been implicated in reproductive toxicity. Gametogenesis, preimplantation embryogenesis and culture are all orchestrated events that determine the success of IVF in human reproductive medicine. This study has delineated the critical concerns of environmental heavy metals and metalloids exposure, impact and outcomes on *in vitro* fertilization in human reproductive medicine. The present review aims to provide a summarized overview of associations between heavy metals and metalloids exposure, IVF outcomes, and reproductive concerns.

## Method

### Information source and database searching

This systematic review was done according to the preferred reporting items for systematic reviews and meta-analyses (PRISMA) guidelines (20). Scientific databases such as Google Scholar, Scopus, EMBASE and PubMed were searched for relevant literature using the following combination of search terms: ‘exposure to heavy metals and metalloids OR ‘toxic trace elements’ and toxicological effects on human reproduction’, ‘heavy metals and reproductive concerns’, ‘heavy metals and reprotoxicity’, ‘*in vitro* fertilization (IVF)’, ‘heavy metals, and metalloids and preimplantation embryogenesis’, ‘heavy metals, and metalloids and gametogenesis in humans’, ‘heavy metals and IVF rate’, ‘heavy metals, and metalloids and pregnancy rate’, ‘heavy metals’ OR ‘toxic trace elements’ and ‘IVF outcome’, ‘effects of toxic/heavy metals and metalloids on reproductive outcome’. Hand-searching of appropriate publications from bibliographies of related international medical societies was also done and retrieved results screened.

### Selection criteria

**Figure 1:**
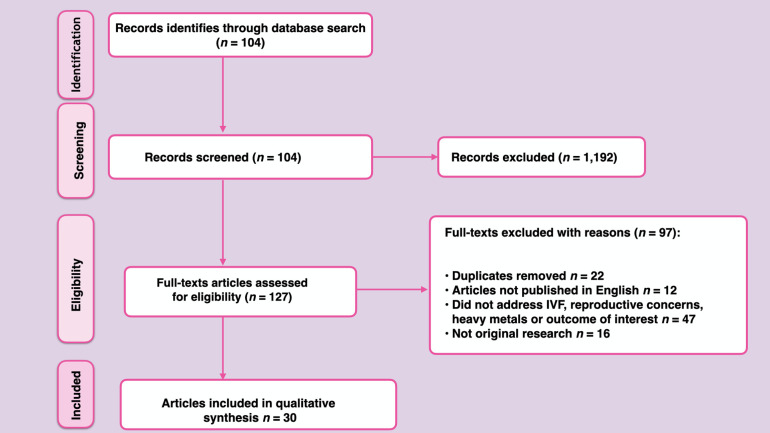
PRISMA flow diagram summarizing systematic search strategy and selection process.

One thousand three hundred and nineteen (1,319) articles were pinpointed from Google Scholar, Scopus, EMBASE and PubMed. After going through abstracts and titles; 1,192 articles were excluded because the studies were irrelevant to the present review. Another 97 articles were excluded with reasons such as: duplicates (22), articles not published in English language (12) or did not address IVF outcome, reproductive concerns, heavy metals/metalloids or outcome of interest (47) or articles were not original research (16). Only 30 articles were eligible and were included in the present study. Taken together, the following guided the inclusion criteria; (1) Human studies that reported sufficient data, study design, sample size, study population, IVF outcomes and reproductive concerns (2) The names of the heavy metals/metalloids implicated were specified. No limits were applied to the year of study of articles included in the review. The PRISMA flow diagram summarizing the selection process is illustrated in Figure 1.

### Quality assessment

The articles that met the inclusion criteria for both IVF outcomes and reproductive concerns were evaluated for quality using a 6-point score designed by the authors, specifically for the present review. Study characteristics listed below, were used for the quality assessment and a score of 0 or 1 was assigned if a particular study met a particular characteristic or not. These include: study design description (if study design was properly described or not, papers were scored 1 or 0), sampling strategy (studies with/without random selection were scored 1/0 respectively, as randomisation guards against selection bias), study population representativeness (if subjects/participants were good representatives of the population or not; papers were scored 1 or 0 respectively), ascertainment of exposure to heavy metals (assessment of heavy metal/metalloid exposure was scored 1 or 0), selection of non-exposed controls (papers were scored 1 or 0, if heavy metal/metalloid exposure was ascertained in participants or not), and appropriate control for variables was also scored 1 or 0 (whether adjustments were made for variables such as age, and socioeconomic status of participants).

## Results

Out of 30 eligible articles included in the present systematic review, 16 articles focused on clinical studies investigating IVF outcomes (Table 1) (16, 21–35) while 14 articles focused on clinical studies that investigated reproductive concerns (Table 2) (36–49).

**Table 1:** Clinical studies on heavy metals/metalloids and IVF outcome.

Heavy metals/metalloids	Study design	Study population/country	Biomarker of exposure	Study duration	IVF outcome	Quality score	References
As, Hg, Cd, Pb	Prospective study	56 women undergoing IVF (USA)	Follicular fluid (FF)	20 months	Association between higher FF Hg concentration and a lower likelihood of biochemical pregnancy (*p* = 0.05) and live birth (*p* = 0.05) Association between higher FF Pb concentration and lower probability of live birth [ (RR) = 0.68, 95% CI: 0.46-1.00; *p* = 0.05]]	5	(35)
Hg, Cd, Pb	Cross-sectional study	58 female patients and 36 male partners (USA)	Blood (Hg and Pb) urine (Cd)	12 months	No associations were detected between blood Hg and fertilization; For female IVF patients, each μg/dl increase in blood Pb level correlated with a 75% reduction in the probability for a retrieved oocyte to be in metaphase-II arrest (relative risk (RR) = 0.25, 95% confidence interval (CI) 0.03–2.50 *p* = 0.240); For male IVF partners, every 1 μg/l increase in urine Cd concentration was correlated with an 81% decrease in the probability for oocyte fertilization (RR = 0.19, 95% CI 0.03–1.35, *p* = 0.097)	5	(21)
Cd	Cross-sectional study	25 female patients and 15 male partners (USA)	Blood	12 months	No association was found for blood Cd from women and oocyte fertilization while, inverse correlation between blood Cd from men	4	(22)
Hg	Prospective pilot study	30 sub-fertile women undergoing IVF (UK)	Hair	18 months	Hair Hg level showed a negative correlation with oocyte yield (*p* < 0.05, β coefficient = 0.38) and follicle number (*p* = 0.03, β coefficient = 0.19) after ovarian stimulation	5	(23)
Hg, Cd, Pb	Cross-sectional study	46 women undergoing IVF (USA)	Follicular fluid (FF)	13 months	An inverse association was found for FF Pb and fertilization (RR = 0.68, *p* = 0.026)	4	(24)
Hg, Pb, Cd	Pilot study	43 women undergoing IVF (USA)	Blood	12 months	Inverse correlation between Pb and IVF outcome Impaired DNA methylation at different CpG sites correlated with exposure to Hg and Pb	4	(16)
Cd, Hg	Cross-sectional study	30 men using IVF (USA)	Seminal plasma	13 months	Increased Pb concentration correlated with about 47% reduction total motile sperm; Negative association was indicated for Hg-adjusted Cd with pregnancy	5	(25)
Hg	Prospective cohort study	205 women undergoing IVF (USA)	Hair	8 years	Levels of Hg in hair samples did not correlate with ovarian stimulation outcomes or rate of fertilization, embryo quality, pregnancy rate or live birth rate.	6	(26)
Pb	Prospective study	101 women undergoing IVF (Turkey)	blood	7 months	Statistically significantly negative correlations between blood Pb levels and number of oocytes, implantation and pregnancy rates	6	(27)
Cr, Mn	Pilot study	58 women undergoing IVF (USA)	Follicular fluid	12 months	FF Cr and Mn correlated negatively with the proportion of mature oocytes	4	(28)
Hg, Pb	Prospective cohort study	194 women undergoing IVF (Spain)	Hair	3 months	Inverse correlation between concentration of Hg in hair and probability of mature oocytes (RR = 0.81, 95% CI: 0.70-0.95); Direct association with Pb (RR = 1.18, 95% CI: 1.03-1.35)	5	(29)
Hg, As, Li, Mo	Case control study	50 women with IVF pregnancy and 158 controls with spontaneous pregnancy and their children (Russia)	Hair	Not Available	Women with IVF pregnancy demonstrated significantly high hair As, Hg, Li levels; Children from IVF pregnancy had significantly higher hair Hg and Mo concentrations compared to control	6	(30)
As	Case control study	33 women with IVF-induced pregnancy (case) and 99 women with natural pregnancy (control) (Russia)	Hair	Not Available	IVF pregnancy correlated significantly with elevated hair levels of As (*p* < 0.05) suggestive of Arsenic overload	5	(31)
Pb, Cd	Cross sectional study	253 couples undergoing IVF (India)	Blood	3 years	No associations were found between IVF outcome and Pb/Cd	6	(34)
Cd, Hg, As, Pb, Cu, Zn, Se, Mg	Cross-sectional study	104 women undergoing IVF (Serbia)	Serum	12 months	Significant correlation of the negative IVF outcome with higher concentrations of Pb (*p* = 0.046) and Cd (*p* = 0.012)	5	(32)
Cr, As, Cd,	Cross-sectional study	103 couples undergoing IVF treatment (China)	Serum and FF samples	5 months	Inverse association between serum Cr level of the female partners with the count of mature oocytes (*p* = 0.033); As levels in female serum and FF were inversely associated with the probabilities to obtain good-quality cleavage embryos (*p* < 0.01); A negative association of FF Cd levels was observed with the probabilities of pregnancy and live birth (*p* = 0.035).	6	(33)

**Table 2:** Clinical studies on heavy metals/metalloids and reproductive concerns.

Heavy metals/metalloids	Study design	Study population/country	Biomarker of exposure	Study duration	Findings	Quality score	References
Al, Mg, Pb	Case-control study	97 pregnant women with offspring were diagnosed with congenital heart defects (CHD group) and 194 pregnant women whose offspring had no CHD (control group) (China)	Umbilical cord blood	2 years	Significantly increased levels of Al, Mg, and Pb during pregnancy correlated with increased risk of CHD in offspring	6	(36)
Pb, Cd, Sb, Ni	Cross-sectional study	206 apparently healthy women administered for prenatal care and monitored for spontaneous abortions (SA) in Iran	Blood	19 months	Only Sb showed a significant positive correlation with the risk of SA (OR: 1.65, 95% CI: 1.08–2.52, *p* value: 0.02)	5	(37)
Pb	Case-control study	300 pregnant women attending a hospital monitored for effects of blood lead levels (BLLs) on spontaneous abortion in China	Blood	2 years	Positive correlations of increased blood Pb levels with spontaneous abortion in five blood lead levels (P1 = 0.64, P2 = 0.02, P3 = 0.01, P4 = 0.02, and P5 = 0.00	5	(38)
Hg	Prospective cohort study	1,204 women in a National Health and Nutrition Examination Survey monitored for Uterine Leiomyomata and Endometriosis (USA)	Blood and urine	5 years	Hg was found positively associated with Uterine leiomyomata (UL) (OR: 1.91, 95% CI: 1.14, 3.25).	5	(39)
Pb, Cd	Prospective cohort study	female workers (*n* = 26,542) who underwent a lead- and cadmium associated special medical examination for endometriosis (EM) (Korea)	blood	3 years	Increased standard admission rate (SAR) (1.24 [95% (CI): 1.03–1.48] and admission odds ratio (OR) [1.44 (95% CI: 1.11–1.85)] in Pb-exposed workers compared to the general population. Co-exposure to lead and cadmium has a synergistic effect with EM.	5	(40)
Cd, Pb, Hg, As	Case control study	33 women with unexplained infertility and 32 fertile women (Turkey)	Endometrial biopsy specimens	20 months	Cd was found in 91% of women with unexplained infertility, compared with 34% of fertile women. Pb was found in 15% of women with unexplained infertility compared with 3% of fertile women. Hg and As were undetected in samples from both groups.	5	(41)
Cd, Cr, Hg, Se, As	Case control	95 pregnant women with history of miscarriage and 100 with no history of miscarriage (control) (China)	Whole blood and urine	2 years	Blood Cd and urine Cr levels were elevated in women with history of miscarriage	5	(42)
Pb, Cd, Hg	Case control	29 pregnant women with history of spontaneous abortion and 20 healthy pregnant women (Turkey)	Blood	8 months	Higher blood Pb levels (*p* = 0.038) were found in the pregnant women with history of spontaneous abortion; no difference in Cd and Hg levels in both groups.	4	(43)
Pb, Cd	Prospective cohort	45 pregnant women diagnosed with threatened abortion and 40 non-pregnant apparently healthy women (Turkey)	Serum	Not available	Significantly increased (*p* < 0.001) serum concentrations of Pb and Cd in cases of threatened abortion	4	(44)
Cd, Pb	Case control	83 women with history of miscarriage and 35 women with no history of miscarriage (control) (Poland)	Blood and fragments of placental tissue	Not available	Higher levels of Cd and Pb in the blood and placenta of pregnant women with miscarriage compared to control	5	(45)
As, Cd, Pb, Hg	Case- control	56 women with PCOS and 50 women without PCOS (control) (Saudi Arabia)	Serum	26 months	Significantly (*p* < 0.001) high levels of Serum As, Cd, Pb, Hg and decreased levels of GSH and SOD in PCOS group compared to the control group -elevated oxidative stress parameters in PCOS women compared to control	5	(46)
Pb, Cd, As, Ba, Hg, U	Prospective cohort study	Male partners (61 infertile and 55 fertile) of infertile women who attended fertility clinic (Lebanon)	Blood and seminal fluid	6 years	Participants with low-quality semen had significantly higher Cd and Ba levels in the seminal fluid compared to participants with normal-quality semen. Significant correlations between low sperm viability and higher blood Cd and B and higher seminal Pb, Cd, Ba, and U	6	(47)
Cd, Pb	Case-control study	35 pregnant women with history of recurrent spontaneous abortion and 35 pregnant women without history of recurrent spontaneous abortion (Nigeria)	*Blood*	17 months	Significant elevation (*p* < 0.05) in Pb, Cd levels in cases compared with control	4	(48)
Cd	Case control	50 women with medically unexplained recurrent spontaneous abortion and 30 healthy fertile women (control group), with no history of spontaneous abortion (Egypt)	*Blood*	Not available	Higher level of cadmium in blood samples of women with unexplained recurrent spontaneous abortion compared to control	5	(49)

## Discussion

### Association of heavy metals/metalloids and *in vitro* fertilization outcome

There is a growing concern regarding the association of environmental heavy metals and metalloids contamination and IVF treatment outcomes. Exposure to heavy metals and metalloids in the environment has no doubt heavily impacted on fertility rate in humans (50, 51). Researchers have found out that overall decline in quality and quantity of the spermatozoa contribute greatly to infertility in men (52). Even with normal semen analysis, some couples undergoing IVF still fail to establish conception (53–55). The numerous external causes of impaired infertility have been linked to exposure to a number of heavy metals and metalloids *via* the alteration of fundamental principle of synchronization between the male and female gametes (56). Heavy metals and metalloids may evoke changes in physiological patterns and processes that elicit mutation by inhibiting DNA, RNA and protein synthesis during spermatogenesis (57). Furthermore, heavy metals and metalloids can induce oxidative stress which generate reactive oxygen species (ROS) (58). ROS generate peroxidation products which can cause depletion of antioxidant systems designed to protect the sperm from oxidative reactions and deterioration of sperm cells (59, 60). High levels of redox activity and aneuploidy rates due to heavy metals and metalloids exposure have been detected in defective spermatozoa and failure on oocyte interaction (61, 62). In females, exposure to heavy metals and metalloids can interfere with ovarian hyper-stimulation and cause poor response, as well as delay of ovulation subsequently contributing to decline in fertilization and conception rate (63).

Exposure to heavy metals and metalloids can trigger epigenetic changes (64) resulting in alteration in gene expression profiles (65). Heavy metals and metalloids such as arsenic and cadmium (66, 67) change DNA methylation in children of prenatally exposed mothers, suggesting that methylation may be a central mechanism by which genomes respond to the environment (66). A study by Hanna and co-workers, demonstrated that a decreased methylation of COL1A2 promoter region correlated with high Pb exposure in women undergoing IVF (16). The COL1A2 gene product is a key component of the uterine cervix and chorio-amniotic membranes (68). Earlier studies have reported that COL1A2 genes are up-regulated in the placental tissue of pregnant tobacco smokers (69). COL1A2 polymorphisms in women have also been associated with preterm prelabour rupture of membranes (which is a leading cause of preterm birth) (68). Therefore, environmentally-induced modifications of COL1A2 gene methylation, might compromise human reproduction (16). Differential DNA methylation with variation enriched at loci within CpG islands were observed in *funiculus umbilicalis* cord blood samples of infants exposed to low-levels of arsenic *in utero* (70). Recently, reports have also associated variation in DNA methylation in the placenta (as well as new-born health outcomes such as growth and neurobehavioral functioning) with different environmental exposures (71–73).

In the present review, 16 articles reported associations between selected heavy metals/metalloids and IVF outcomes in different human samples such as hair, follicular fluid, whole blood, serum, urine and seminal fluid. Heavy metals and metalloids investigated in these studies include Pb, Hg, Cd, Cr, Mn, and As.

#### Heavy metals and metalloids in hair samples

Hair analysis provides a novel approach of probing the impact of long-term exposure to heavy metals and metalloids on reproductive outcomes (74, 75). Five studies (23, 26, 29–31) investigated heavy metals and metalloids in hair samples of women undergoing IVF. In a study to examine the heavy metals and metalloids status in hair samples of women with IVF pregnancy and their 9-month-old children, Skalny et al. (29) found out that women with IVF pregnancy demonstrated significantly high hair As, Hg, Li levels and their children also had significantly elevated hair Hg and Mo concentrations compared to control (30). Similarly, another case-control study by Skalny et al. (30) demonstrated that IVF pregnancy correlated significantly with elevated hair levels of As (*p* < 0.05) suggestive of arsenic overload in IVF patients (31). Garcia-Fortea et al. (28) in a prospective cohort study examined 194 women undergoing IVF and found an inverse correlation between concentration of Hg in hair and probability of mature oocytes (RR = 0.81, 95% CI: 0.70–0.95) while a direct association was demonstrated with Pb (RR = 1.18, 95% CI: 1.03–1.35) (29). Similarly, an earlier study by Dickerson et al. (22) revealed a negative correlation between hair Hg level with oocyte yield (*p* < 0.05, β coefficient = 0.38) and follicle number (*p* = 0.03, β coefficient = 0.19) following ovarian stimulation (23). Wright et al. (25) found no association between hair Hg concentrations with ovarian stimulation outcomes (total and mature oocyte yields, peak estradiol levels), fertilization rate, clinical pregnancy rate, embryo quality, or live birth rate (26).

#### Heavy metals and metalloids in whole blood samples

Blood, serum and urine sampling provide indications of short-term changes in heavy metal and metalloids levels (30). Heavy metals and metalloids concentrations in blood and urine are the most widely used biomarkers to document adverse health effects (29).

Five studies (16, 21, 22, 27, 34) investigated the associations between heavy metals and metalloids in whole blood samples and *in vitro* fertilization (IVF) outcomes. A study by Kim et al. (2010) in 25 female patients and 15 male partners showed no association between blood Cd in women and oocyte fertilization while, inverse correlation was found between blood Cd in men (22). Similarly, a later study by Kumar et al. (33), also showed no association between maternal and paternal blood Cd, Pb levels and IVF outcome (34). Bloom et al. (20) found no associations between blood Hg and fertilization; 1 μg/dl increase in blood Pb level correlated with a 75% reduction in the probability for a retrieved oocyte to be in metaphase-II arrest (relative risk (RR) = 0.25, 95% confidence interval (CI) 0.03–2.50, *p* = 0.240) (21). A study by Tolunay et al. (26) showed negative correlations between blood Pb concentrations and number of metaphase II (MII) oocytes, implantation, pregnancy rates (27). This data is consistent with results obtained by Hanna et al. (15) in which an inverse correlation between Pb and IVF outcome was obtained. In addition, impaired DNA methylation was also observed at different CpG sites which correlated with Hg and Pb exposure (16).

#### Heavy metals and metalloids in urine samples

A study by Bloom et al. (20) investigated impact of heavy metals and metalloids in male partners of IVF patients. Their results showed that every 1 μg/L increase in urine Cd concentration correlated with an 81% decrease in the probability for oocyte fertilization (RR = 0.19, 95% CI 0.03–1.35, *p* = 0.097) (21).

#### Heavy metals and metalloids in seminal plasma

Kim et al. (24) measured heavy metals in seminal plasma of men using IVF to probe associations between semen quality and IVF outcomes. While increased Pb concentration correlated with about 47% reduction in total motile sperm, a negative association was indicated for Hg-adjusted Cd with pregnancy (25).

#### Heavy metals and metalloids in serum samples

Two studies (32, 33) investigated the associations between heavy metals and metalloids in serum and *in vitro* fertilization (IVF) outcomes. A study by Wu et al. (32) revealed an inverse association between serum Cr level of IVF patients with the count of mature oocytes (*p* = 0.033), in addition, arsenic (As) concentration in female serum were inversely associated with the probabilities to obtain good-quality cleavage embryos (*p* < 0.01) (33). On the other hand, Tulic et al. (31) found a significant negative correlation between IVF outcome with higher serum concentrations of Pb (*p* = 0.046) and Cd (*p* = 0.012) (32).

#### Heavy metals and metalloids in follicular fluid (FF)

Follicular fluid (FF) samples provide additional information on the risk of infertility in women (29). However, it has a limitation of not being easily accessible and highly invasive, therefore, the use of other biomarkers are usually considered, especially when sampling does not pose any risk to patients, in addition to being performed at a minimal cost (23). Four studies (24, 28, 33, 35) investigated the associations between heavy metals and metalloids in follicular fluid and *in vitro* fertilization (IVF) outcomes. A cross-sectional study by Bloom et al. (23) conducted in 46 women undergoing IVF revealed an inverse association between FF Pb concentration and fertilization (RR = 0.68, *p* = 0.026) (24). Ingle et al. (27) found a negative correlation between FF Cr and Mn levels with the proportion of mature oocytes (28). Wu et al. (32) noted that As levels in FF samples were inversely associated with the probabilities to obtain good-quality cleavage embryos (*p* < 0.01) and a negative association of FF Cd levels found with the probabilities of pregnancy and live birth (*p* = 0.035) (33). A more recent study by Butts et al. (34) investigated the association between follicular fluid (FF) concentrations of As, Hg, Pb and Cd with IVF outcomes among women undergoing IVF. Their results showed lower probabilities of biochemical pregnancy (*p* = 0.05) and live birth (*p* = 0.05) at follicular fluid Hg concentrations greater than 0.51 µg/L Hg. In addition, higher follicular fluid Pb concentrations also correlated with a lower probability of live birth (RR = 0.68, 95% CI: 0.46–1.00; *p* = 0.05) (35).

### Heavy metals/metalloids and reproductive concerns

Environmental contamination with heavy metals and metalloids has become a major area of public health concern especially for women of childbearing age, as it can cause infertility and reproductive dysfunction (76–79). The interference of heavy metals and metalloids on human reproduction ranges from uterine leiomyomata, spontaneous abortions, polycystic ovary syndrome (PCOS), birth defects, endometriosis, abnormal semen quality and functionality, impaired embryogenesis, as well as stillbirths (46, 75, 79–81). Recent reports have highlighted the endocrine-disrupting effect of heavy metals and metalloids on the pituitary ovarian axis indicative of their potential associations with female reproductive health (82, 83). Heavy metals and metalloids may trigger hormonal changes that alter the menstrual cycle, ovulation, and female fertility (84). Several studies have investigated the role of heavy metals and metalloids in altering hormonal levels (85, 86). For instance, a study by Chang (83) reported that women with blood Pb levels higher than 25 μg/L had a threefold increased risk of infertility compared to women with blood Pb levels less than 25 μg/(87). Similarly, another study reported that for every 1 μg/L elevation in Cd levels, a 21% increase in the levels of early follicular phase oestradiol (E2); serum follicle stimulating hormone (FSH) and luteinizing hormone (LH) concentrations were noted (88). Taken together, high levels of reproductive anomalies in women of reproductive age have driven the course of assisted reproduction such as *in vitro* fertilization.

In the present review, 14 studies investigated the impact of exposure of toxic metals on reproductive concerns. Two studies investigated the role of heavy metals and metalloids in infertility (41, 47). The study by Tanrikut et al. (40) revealed that Cd was found in 91% of women diagnosed with unexplained infertility against 34% of fertile women. Pb was found in 15% of women diagnosed with unexplained infertility and 3% of fertile women. Hg and As were undetected in endometrial samples from either groups of women. Results obtained suggested Cd to be a major contributing factor in the aetiology of unexplained infertility (41). Sukhn et al. (46) examined the impact of heavy metals and metalloids in male partners of infertile women attending a fertility clinic. Their data showed significant (*p* < 0.05) associations between low sperm viability and higher blood Cd and Ba, as well as higher seminal Pb, Cd, Ba, and U (47).

One study investigated association of heavy metals with congenital heart defects (CHD) in offspring (36). In that study, high levels of Al, Mg, and Pb in umbilical cord blood significantly correlated with increased risk of CHD in offspring Al ^a^OR (adjusted odds ratio) = 4.22, 95% CI: 1.35–13.16, *p* = 0.013), Mg (^a^OR = 8.00, 95% CI: 1.52–42.08, *p* = 0.014), and Pb (^a^OR = 3.82, 95% CI: 0.96–15.23, *p* = 0.049) (36).

Zhang et al. (38) probed the role of Hg in uterine leiomyomata (UL) (39). Data from that study revealed that Hg was found to be positively associated with UL (OR: 1.91, 95% CI: 1.14, 3.25).

Abudawood et al. (45) investigated the impact of heavy metals and metalloids in pregnant women diagnosed with PCOS (46). The role of heavy metals and metalloids in generating oxidative stress (an etiological factor in PCOS) was examined in that study. Results obtained from that study demonstrated high levels of serum As, Cd, Pb, and Hg which correlated with significantly diminished levels of serum glutathione (GSH) and superoxide dismutase (SOD) in the PCOS group compared to the control group at *p* < 0.001 (46).

Eight studies investigated association of heavy metals and metalloids with miscarriage/spontaneous abortions (37, 38, 42–45, 48, 49). Ajayi et al. (47) found significant increase (*p* < 0.05) in the serum Pb, and Cd in pregnant women with history of recurrent spontaneous abortion compared with controls (48). The study by Vigeh et al. (37) revealed that out of all the metals and metalloids evaluated, only Sb showed a significant positive correlation with the risk of spontaneous abortions (OR: 1.65, 95% CI: 1.08–2.52, *p* value: 0.02). Ou et al. (37) evaluated effects of blood lead levels (BLLs) on spontaneous abortion. The mean BLLs in both case and control groups were 27.17 μg/L and 17.28 μg/L respectively (*p* = 0.000). The odds ratios for spontaneous abortion in five blood lead levels (5–9, 10–14, 15–24, 25–39, and ≥40 μg/L) were 1.58 (0.23–10.90), 3.13 (2.11–9.08), 4.63 (1.45–14.83), 6.33 (1.95–20.56), and 22.56 (4.91–103.66), respectively, indicative of a significant trend (P1 = 0.64, P2 = 0.02, P3 = 0.01, P4 = 0.02, and P5 = 0.00) (38). Yildrim and Derici (42) found higher blood Pb levels (*p* = 0.038) in pregnant women with history of spontaneous abortion compared to control (43), while the study by Turan et al. (43) showed significantly increased (*p* < 0.001) serum concentrations of Pb and Cd in women with threatened abortion compared to control (44). Jie et al. (41) demonstrated that blood cadmium >0.4 µg/L or urine chromium >2 µg/L in pregnant women was indicative of a higher risk of spontaneous abortion (42). Omeljaniuk et al. (44) examined the role of Pb and Cd in women who had miscarriage. The mean concentrations of Pb (35.54 ± 11.0 µg/L) and Cd (2.730 ± 2.07 µg/L) in the blood of women who had miscarriage was higher compared to control (Cd 1.035 ± 0.59 µg/L; Pb 27.11 ± 4.6 µg/L). on the other hand, the mean concentrations of Pb (199.6 ± 348 µg/L) and Cd (214.4 ± 514 µg/L) in the placenta of women who had miscarriage was higher compared to control (Cd 127.4 ± 85 ng/L; Pb 26.35 ± 7.9 ng/L) (45). Similarly, Saad et al. (48) found higher levels of Cd in women who miscarried accompanied with increased levels of plasma malondialdehyde and decreased levels of antioxidants glutathione and glutathione peroxidase suggestive of oxidative stress as a major etiological factor (49). In these 8 studies, Pb and Cd stand out as major reoccurring metals in women with history of spontaneous abortion.

Two studies investigated the relationship between heavy metals/metalloids and endometriosis (39, 40). Zhang et al. (38) found no association between Hg and endometriosis (39) while Kim et al. (39) observed an increase in the standard admission rate (SAR) (1.24 (95% CI: 1.03–1.48) and admission odds ratio (OR) [1.44 (95% CI: 1.11–1.85)] for endometriosis in Pb exposed workers compared with that of the general population (40).

## Conclusion

Chronic environmental exposure to heavy metals and metalloids can alter the function of sexual accessory tissues as well as reproductive hormones in humans. These delay the timecourse of sexual maturation directly or indirectly thereby affecting reproductive competence in males and females. Data collated in this review demonstrate that heavy metals and metalloids in body samples of women undergoing IVF correlated negatively with oocyte fertilization/pregnancy rates. Similarly, high concentrations of these metals were also associated with infertility, spontaneous abortion/miscarriage, congenital heart disease, PCOS, endometriosis as well as uterine leiomyomata. Therefore, couples undergoing IVF and those of reproductive age may be screened for heavy metals and metalloids load. In addition, therapeutic strategies to reduce body metals and metalloids burden should be undertaken to enhance pregnancy outcomes.

## Data Availability

The original contributions presented in the study are included in the article/Supplementary Material, further inquiries can be directed to the corresponding author/s.
